# Vav2 is a novel APP-interacting protein that regulates APP protein level

**DOI:** 10.1038/s41598-022-16883-z

**Published:** 2022-07-26

**Authors:** Youjia Zhang, Xiaxin Yang, Yongrui Liu, Liang Ge, Jiarong Wang, Xiulian Sun, Bo Wu, Junfeng Wang

**Affiliations:** 1 High Magnetic Field Laboratory, Key Laboratory of High Magnetic Field and Ion Beam Physical Biology, Hefei Institutes of Physical Science, Chinese Academy of Sciences, Hefei, Anhui China; 2 University of Science and Technology of China, Hefei, Anhui China; 3 Department of Neurology, Qilu Hospital of Shandong University, Jinan, China; 4 Brain Research Institute, Qilu Hospital of Shandong University, Jinan, China; 5 The Key Laboratory of Cardiovascular Remodeling and Function Research, Chinese Ministry of Education, Chinese National Health Commission, Qilu Hospital of Shandong University, Jinan, China; 6 NHC Key Laboratory of Otorhinolaryngology, Qilu Hospital, Cheeloo College of Medicine, Shandong University, Jinan, Shandong China; 7 Institute of Physical Science and Information Technology, Anhui University, Hefei, Anhui China

**Keywords:** Structural biology, NMR spectroscopy, X-ray crystallography, Molecular neuroscience, Biochemistry, Neuroscience, Phosphorylation, Alzheimer's disease

## Abstract

Amyloid precursor protein (APP) is a transmembrane protein that plays critical role in the pathogenesis of Alzheimer's disease (AD). It is also involved in many types of cancers. Increasing evidence has shown that the tyrosine phosphorylation site Y682 in the intracellular tail of APP is crucial for APP function. Here, we report that Vav2, a guanine nucleotide exchange factor (GEF) for Rho family GTPase, is a novel interaction partner of APP. We found that Vav2-SH2 domain was able to bind directly to the Y682-phosphorylated intracellular tail of APP through isothermal titration calorimetry and NMR titrating experiments. The crystal structure of Vav2-SH2 in complex with an APP-derived phosphopeptide was determined to understand the structural basis of this recognition specificity. The interaction of APP and Vav2 in a full-length manner was further confirmed in cells by GST pull-down, co-immunoprecipitation and immunofluorescence staining experiments. In addition, we found overexpression of Vav2 could inhibit APP degradation and markedly increase the protein levels of APP and its cleavage productions in 20E2 cells, and this function of Vav2 required a functional SH2 domain.

## Introduction

Amyloid precursor protein (APP) is a type-I transmembrane protein that is crucial for neuronal development and homeostasis^1,2^. It is best known for its role in the pathogenesis of Alzheimer’s disease (AD)^3^. AD is a neurodegenerative disease and is the most often found form of dementia among elderly people. Currently, there is no effective treatment for this disease^4^. The pathological hallmark of AD is the presence of amyloid plaques in the brain of AD patients^5,6^. APP is proteolytically cleaved by β- and γ-secretase to generate the amyloid β peptide (Aβ), which is the major component of amyloid plaques^7–9^. In addition, APP has been found to be overexpressed in various cancers, including breast cancer and can promote cancer cell migration and invasion^1,10,11^.

In human, there are three main isoforms of APP derived by alternative splicing: a 695-residue form expressed primarily in the central nervous system and 751- and 770-residue forms that are ubiquitously expressed^12^. All three forms share a similar architecture with a large ectodomain, a single transmembrane domain and a short intracellular tail^13,14^. The short intracellular tail of APP is essential for APP function^15,16^. Numerous cytosolic proteins are found to bind directly to the intracellular domain of APP^17^. It is 47 amino acids in length and contains several functional motifs and phosphorylation sites, including Y682, whose phosphorylation level has been shown to be significantly increased in AD brains^18,19^. Most interactions of APP involve the Y682 phosphorylation site. Y682 modulates the interactions of APP with different proteins through its phosphorylation and dephosphorylation^20^. Some proteins, such as X11^21–23^, Fe65^24–26^, and JIP-1^27^ interact with APP when Y682 is not phosphorylated, whereas others, such as Grb2^28,29^, Shc^30^, only when Y682 is phosphorylated. Increasing evidences show that these interactions play key roles in the regulation of APP processing and function, and in AD^15,31–35^. For example, Mint and Fe65 family modulate cellular trafficking and the processing of APP and affect Aβ production^36,37^. JIP-1 facilitates APP axonal trafficking and regulates APP-dependent axonal transport of synaptic vesicles^38^. Notably, a recent study showed that targeting the APP-Mint2 protein–protein interaction with a peptide-based inhibitor reduced amyloid β formation, which may present an alternative strategy in the pursuit of new therapeutic approaches in AD treatment^39^.

Herein, we report the identification of Vav2 as a novel interaction partner for APP. It is a guanine nucleotide exchange factor (GEF) for Rho GTPases belonging to the Vav family (Vav1, Vav2 and Vav3)^40,41^. Vav2 is broadly expressed in human tissues and is involved in regulating various biological processes, including cell spreading and migration, neuronal development, angiogenesis, and cancer cell motility^42–47^. Vav2 consists of multiple domains, including a calponin homology (CH) domain, an Acidic (Ac) region, a catalytic Dbl homology (DH), a pleckstrin homology (PH) domain, a zinc finger (ZF) domain, a Src homology 2 (SH2) domain and two Src homology 3 (SH3) domains^48^. Among all Ras superfamily GEFs, only Vav family proteins possess an SH2 domain, a common protein interaction module that specifically recognizes phosphotyrosine motif^49,50^. Through its SH2 domain, Vav2 can bind to the tyrosine-phosphorylated cytoplasmic domains of several membrane receptors and then mediate different extracellular signals to intracellular responses^51–53^.

Our research demonstrated that Vav2 can interact with APP through its SH2 domain which binds directly to the Y682-phosphorylated APP intracellular tail. A crystal structure of Vav2-SH2 domain in complex with the APP-derived phosphopeptide APP-pY682 (QNG-pY-ENPT, residues 679–686 of APP695) was determined at 2.45 Å resolution, which revealed a conserved recognition mechanism. The interaction of APP and Vav2 in a full-length manner was further confirmed by GST pull-down experiments, co-immunoprecipitation and immunofluorescence staining. Moreover, we found that overexpression of Vav2 significantly increased APP protein level and promoted Aβ40 generation in 20E2 cells, an AD cell model. We further show that Vav2 overexpression inhibited APP protein degradation. This function of Vav2 requires its SH2 domain. Together, these findings uncover a novel interaction between Vav2 and APP and a regulatory role of Vav2 in APP turnover.

## Results

### Identification and characterization of a direct interaction between Vav2-SH2 domain and Y682-phosphorylated APP peptide

**Figure 1:**
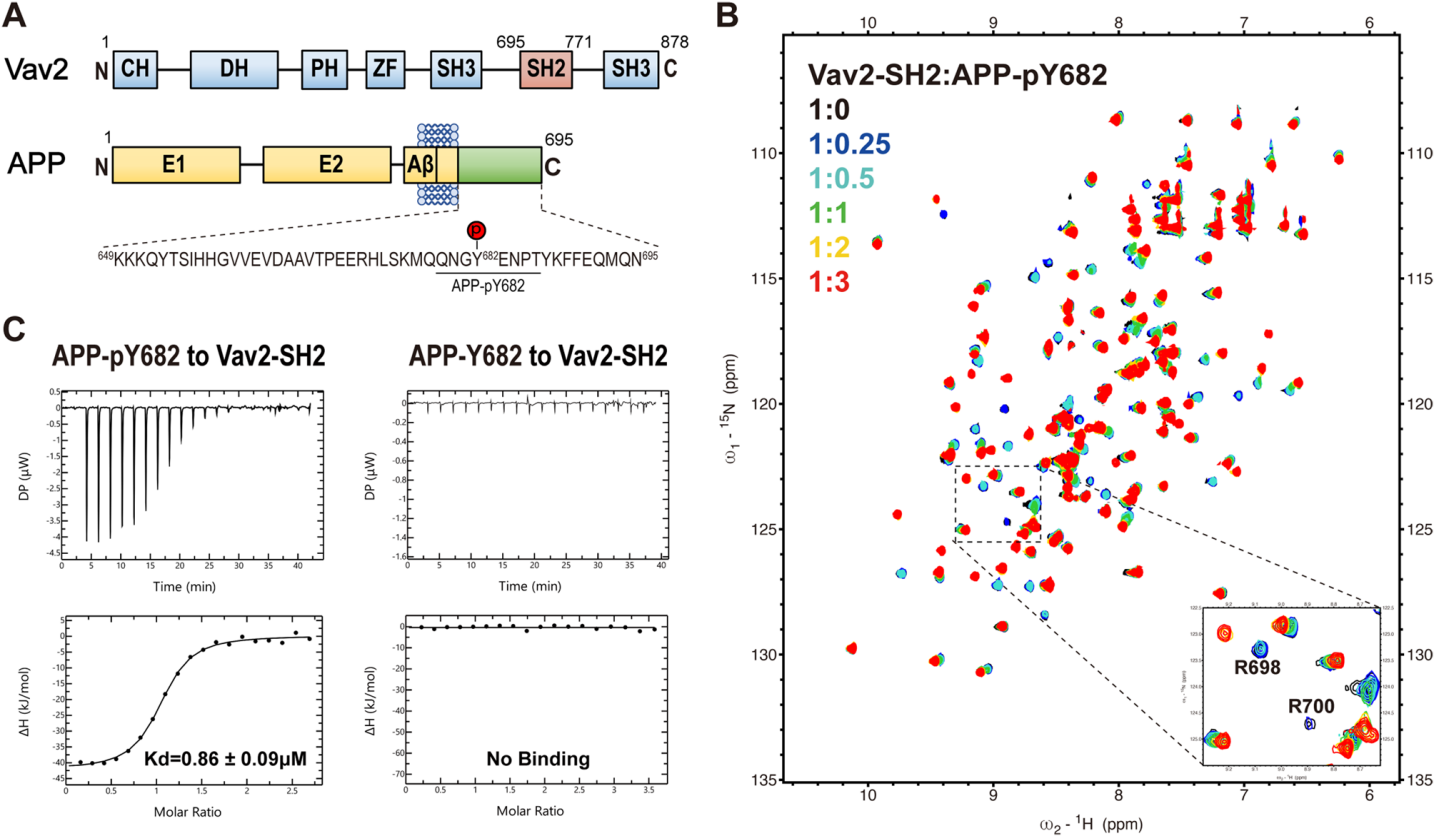
Binding of Vav2-SH2 to phosphotyrosine peptide APP-pY682 derived from APP. (**A**) Schematic representation of human Vav2 and APP. Sequence of APP intracellular domain and phosphotyrosine peptide APP-pY682 are labeled. (**B**) Overlay of ^1^H–^15^N HSQC spectra of Vav2-SH2 in the absence (black) and in the presence of increasing amounts of the peptide APP-pY682 (QNG-pY-ENPT). The molar ratios of the protein to the peptide are shown in the inset: 1:0 (black), 1:0.25 (blue), 1:0.5 (cyan), 1:1.0 (green), 1:2.0 (yellow) and 1:3.0 (red). Residues R698 and R700 are zoomed in. (**C**) ITC measurements of the binding affinity of APP-pY682 and APP-Y682 to Vav2-SH2.

Our previous work showed that Vav2-SH2 domain prefers mostly to recognize a consensus motif of pY-E-X-P, where X denotes any amino acid^54,55^. We found that the tyrosine phosphorylation site Y682 in the intracellular domain of APP, within the sequence of YENP, exactly matched this consensus motif. Therefore, we speculated that Y682-phosphorylated APP might be able to bind the SH2 domain of Vav2. To explore the possibility, we performed NMR titration and isothermal titration calorimetry (ITC) experiments using the recombinantly expressed Vav2-SH2 protein and a synthesized phosphotyrosine peptide derived from residue Y682 in APP (termed APP-pY682: QNG-pY-ENPT, residues 679–686 of APP695) (Fig. 1A and Table S1). As a control, the non-phosphorylated form of this peptide (referred to as APP-Y682) was also included. The purified Vav2-SH2 protein was determined by SDS-PAGE with high purity (> 98%) (Fig. S1), and the final protein yields were about 15 mg and 10 mg per L culture for the unlabeled and ^15^N-labeled protein, respectively.

For NMR titrating experiments, 2D ^1^H–^15^N HSQC spectra^56^ of ^15^N-labeled Vav2-SH2 domain at a series of protein to peptide ratios were collected, respectively. As shown in Fig. S2, titration with the non-phosphorylated APP peptide did not induce any chemical shift perturbations in the ^1^H–^15^N HSQC spectra of Vav2-SH2 domain. In contrast, titration with the phosphopeptide APP-pY682 caused substantial chemical shift perturbations (CSPs) in the protein, indicating direct binding (Fig. 1B). During the titration, many resonances corresponding to the free state of Vav2-SH2 disappeared, while another set of crosspeaks, corresponding to the bound state appeared. All these residues were mapped on the structure of Vav2-SH2 (Fig. S3). This pattern of CSPs demonstrated the formation of a complex that is in slow to intermediate exchange on the chemical shift NMR time scale. ITC reported a binding Kd of 0.86 μM for Vav2-SH2 in complex with APP-pY682 peptide and no binding of Vav2-SH2 and APP-Y682 (Fig. 1C).

### Crystal structure of Vav2-SH2 domain in complex with APP-pY682 peptide

**Figure 2:**
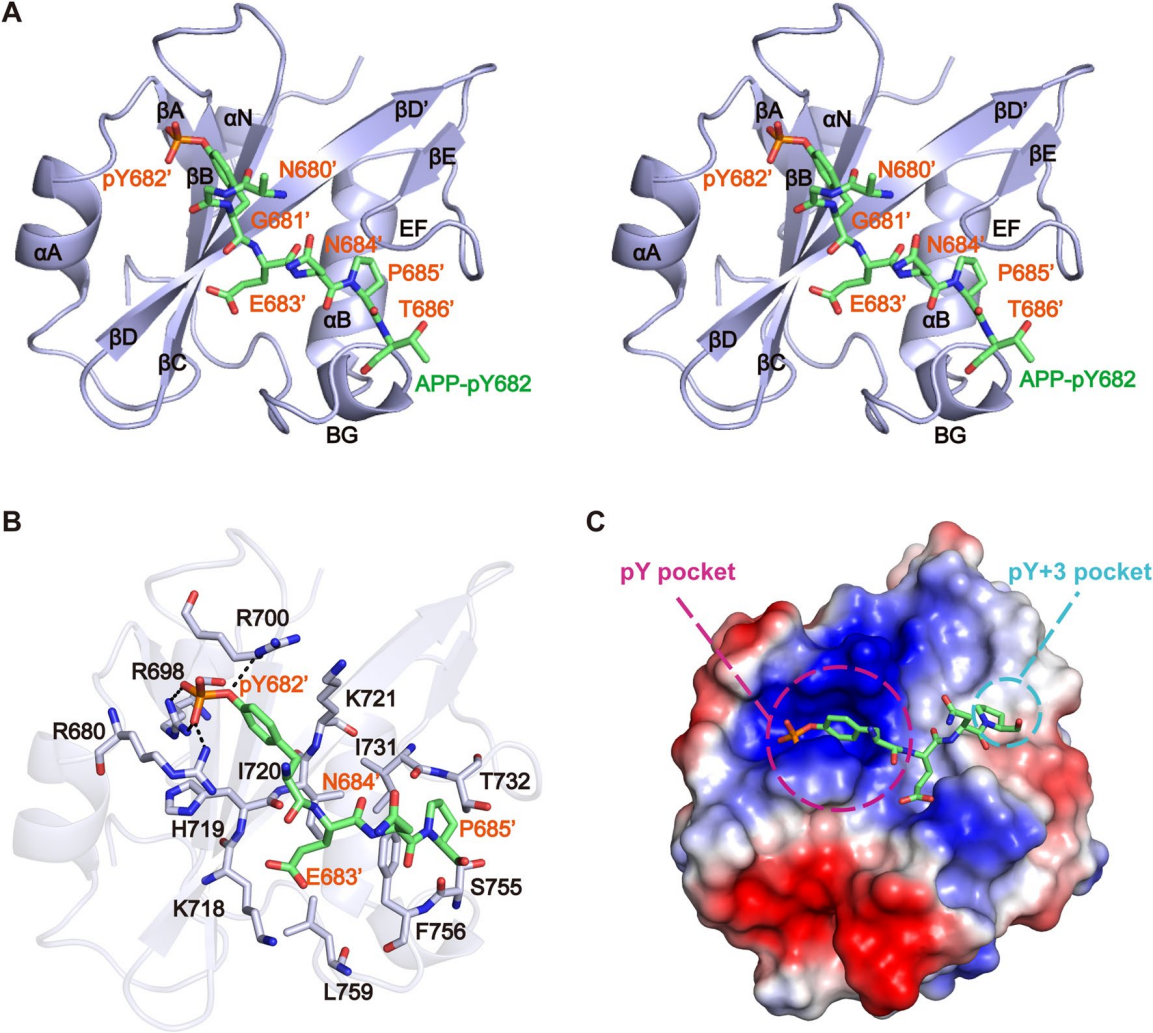
Specific interaction of the Vav2-SH2 domain and the peptide APP-pY682. (**A**) Overall stereoview of Vav2-SH2 domain in complex with peptide APP-pY682. Vav2-SH2 is colored purple, with the secondary structures are labeled. The APP-pY682 peptide (green) is shown in stick mode, with the residues N680′, G681′, pY682′, E683′, N684′, P685′ and T686′ are labeled. (**B**) Detailed interactions of the peptide APP-pY682 with SH2 domain. The peptide APP-pY682 and the side chains of crucial residues are shown in stick mode and labeled respectively. Selected hydrogen bonds are colored in black. (**C**) Electrostatic potential surface representation of Vav2-SH2 domain bound to APP-pY682 peptide (green). The pY and pY + 3 pockets are indicated by magenta and blue circles separately.

To further understand the structural basis for the specific recognition of Y682-phosphorylated APP by Vav2-SH2 domain, we solved the crystal structure of Vav2-SH2 in complex with the APP-pY682 peptide. The structure was refined to 2.45 Å resolution (Fig. 2 and Table 1) (PDB entry: 7WFY).

**Table 1:** Data collection and refinement statistics.

	Vav2-SH2/APP-pY682
**Data collection**	
Wavelength (Å)	0.979
Space group	C222
Cell dimension	
a, b, c (Å)	62.743, 109.589, 41.369
α, β, γ (°)	90, 90, 90
Resolution* (Å)	50.00–2.45 (2.49–2.45)
Rmerge (%)	8.5 (34.7)
I/σI	26.33 (8)
Completeness (%)	100 (100)
Redundancy	12.7 (11.4)
**Refinement**	
No. reflections used/free	5421/583
Resolution range (Å)	41.37–2.45
*R*_work_/*R*_free_ (%)	22.75/26.78
R.m.s. deviations	
Bond lengths (Å)	0.008
Bond angles (°)	0.995
*B*-factors (Å^2^)	
Protein	41.57
Water	39.89
No. atoms	
Protein	928
Water	8
Ramachandran plot	
Favored/allowed/outlier (%)	96.19/3.81/0

In the complex, the general fold of Vav2-SH2 domain is almost the same with that observed in its free state^55^, which consists of a short N-terminal α-helix (αN), a central β-sheet (βB–βD), two α-helices (αA and αB) and a small β-sheet (βD’ and βE) (Fig. 2A). The peptide lies in an extended backbone conformation roughly perpendicular to the central β-strands of the SH2 domain (Fig. 2A,B). Phosphotyrosine pY682′ inserts into the canonical pY-binding pocket formed by R680, R698, R700, H719 and K721 (Fig. 2B,C). The phosphate moiety of pY682′ forms a hydrogen bond network with the side chains of R680, R698 and R700. The aromatic moiety of this residue is packed against residues H719 and K721. Residue E683′ (pY + 1) makes hydrophobic interactions with the side chains of residues K718, I720, F756 and L759. This residue is further stabilized by forming a hydrogen bond between its backbone amide nitrogen and carbonyl oxygen atom of residue H719. Residue N684′ (pY + 2) does not interact with SH2 domain. Residue P685′ (pY + 3) patches on the surface of the pY + 3 pocket formed by EF and BG loops and makes contacts with T732, S755 and F756 (Fig. 2B,C). Other residues of the APP-pY682 peptide show little or no interaction with the SH2 protein.

### Comparison of the recognitions of Y682-phosphorylated APP by Vav2-SH2 and Grb2-SH2

**Figure 3:**
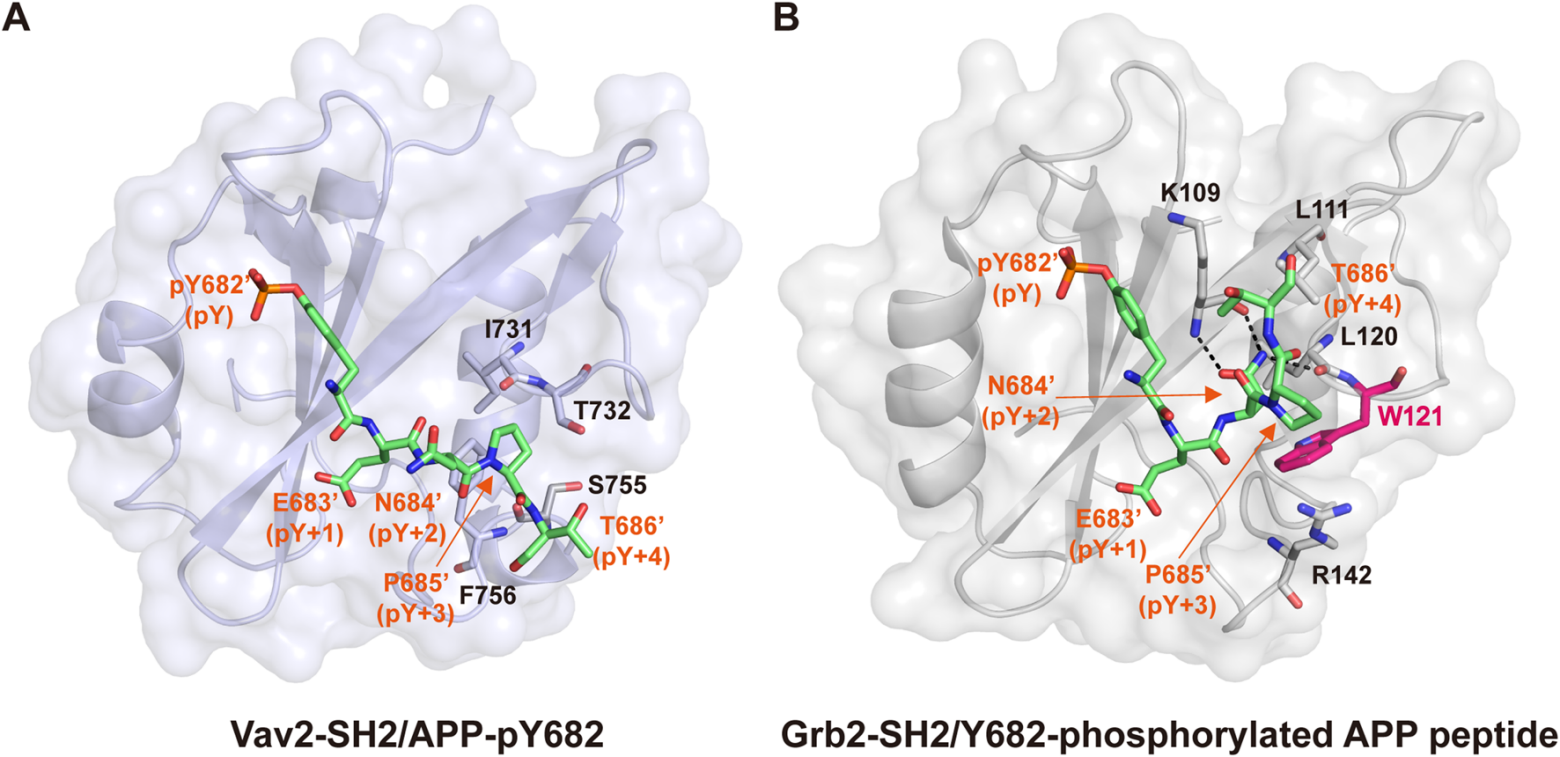
Comparison of the phosphopeptide-binding sites in Vav2-SH2 and Grb2-SH2. (**A**,**B**) The presentation of the structures of Vav2-SH2 complexed with APP-derived phosphopeptide (^682^pY-ENPT^686^) and Grb2-SH2 (PDB code: 3MXC). Vav2-SH2 domain (in purple) and Grb2-SH2 domain (in grey) are shown in ribbon and surface representation, APP-derived phosphopeptide (in green) and the side chains of crucial residues is shown in stick representation. Conformation of APP-pY682 as seen in complex with Vav2-SH2 domain is hindered by W121 (in red) of EF loop and BG loop when bound to the Grb2-SH2 domain. Selected hydrogen bonds are colored in black.

The SH2 domain of Grb2 can also bind to Y682-phosphorylated APP. The crystal structure of Grb2-SH2 in complex with a Y682-phosphorylated APP peptide was reported previously^29^. Structure comparison reveals a significant difference in the mechanisms of the peptide binding to the SH2 domains of Vav2 and Grb2. As shown above and in Fig. 3A, the APP-pY682 peptide binds to Vav2-SH2 in an extended conformation. However, when bound to Grb2-SH2 domain the peptide adopts a U-shape conformation (Fig. 3B). This is due to the presence of a bulky Tryptophan residue (W121) in the EF loop of Grb2-SH2, which is a Threonine residue (T732) at the corresponding position of Vav2-SH2. The large sidechain of W121 occupies the pY + 3 pocket and sterically hinders the phosphopeptide from assuming an extended conformation and forces it into the U-shape conformation. In the complex of Grb2-SH2 with pY682-phosphorylated APP peptide, both residues N684′ (pY + 2) and T686′ (pY + 4) of APP interact strongly with the protein. N684′ forms a network of hydrogen bonds with K109 and L120, while T686′ interacts with L111 and K109 in Grb2-SH2. In contrast, in the complex of Vav2-SH2 with APP-pY682, neither N684′ nor T686′ contacts with the protein.

### Full-length APP and Vav2 interact in mammalian cells

**Figure 4:**
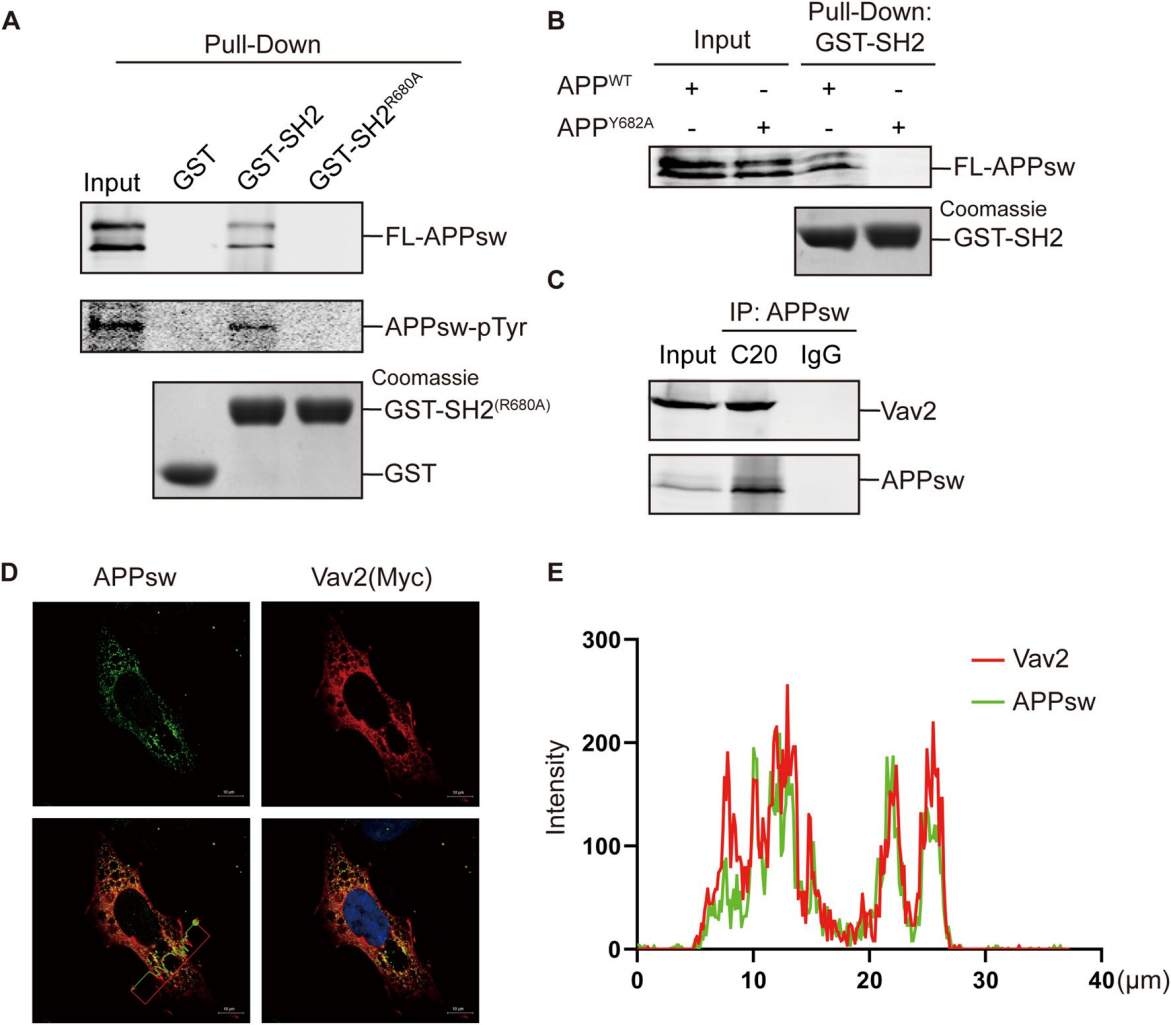
Interaction of Vav2 with full-length APP in cells. (**A**) GST pull-down assay of APP from lysate of cells stable expressing APP (20E2 cells) with the purified GST fusion proteins. GST fusion proteins were detected by Coomassie blue staining. FL-APPsw were detected by western blot using C20 antibody and the Tyr-phosphorylated APP were detected by western blot using anti-Phospho-Tyrosine antibody. Original blots and gels are presented in Supplementary Figures. (**B**) GST pull-down assay of the wild type and mutant of APP with purified GST-SH2 fusion proteins. Original blots and gels are presented in Supplementary Figures. (**C**) Co-IP was performed using HEK293 cells transfected with Vav2-Myc and pAPPsw. C20 was used as the pull-down antibody and anti-myc antibody was used as detection antibody. Original blots are presented in Supplementary Figures. (**D**) Vav2 co-localizes with APP in HEK293 cells. HEK293 cells transfected with Vav2-Myc and pAPPsw were stained with anti-myc (red) and C20 (green) antibodies, DAPI (blue) was used to indicate the nucleus. Images were captured by LSM 880 fluorescent confocal microscope. The line with an arrow in the third picture indicates the selected line used in co-localization analysis (right).

To determine whether the SH2 domain of Vav2 can interact with full-length APP in a cellular environment, we purified wild-type GST-Vav2-SH2 protein and its R680A mutant (as a negative control) and then performed GST pull-down assays^55^. ITC experiments showed that mutation of R680 to Ala in Vav2-SH2 domain nearly abolished its binding with APP-pY682 (Fig. S4). In the GST pull-down assays, we used lysates from 20E2 cells. Immunoprecipitation of APPsw and immunoblotting with an anti-phosphotyrosine antibody revealed that APPsw is tyrosine phosphorylated in 20E2 cells (Fig. S5). The GST pull-down assays showed that wild-type GST-Vav2-SH2, but not GST alone or the R680A mutant bound selectively to full-length APPsw (Fig. 4A). To investigate whether the tyrosine phosphorylation site Y682 of APP is involved in binding with Vav2-SH2 domain, we generated an Y682A mutant of APPsw (APPsw^Y682A^). GST pull-down assays were performed to test the binding of APPsw^Y682A^ to Vav2-SH2 domain using HEK293 cells lysate expressing APPsw^Y682A^. HEK293 cells lysate expressing wild type APPsw was used as a positive control. As expected, the results showed that APPsw^Y682A^ abolish its binding to Vav2-SH2 (Fig. 4B). To test whether the interaction between full-length APPsw and Vav2 occurs in cells, a co-immunoprecipitation experiment was performed. HEK293 cells were transiently co-transfected with pAPPsw and myc-tagged Vav2 plasmids. As shown in Fig. 4C, Vav2 was efficiently precipitated by an antibody against APPsw (C20), but not by the control IgG. Moreover, immunofluorescence staining experiments show that APPsw were co-localized with Vav2 (Fig. 4D). Taken together, these results indicated that Vav2 can interact with APP through its SH2 domain in mammalian cells.

### Overexpression of Vav2 markedly increases APP protein level and Aβ40 generation

**Figure 5:**
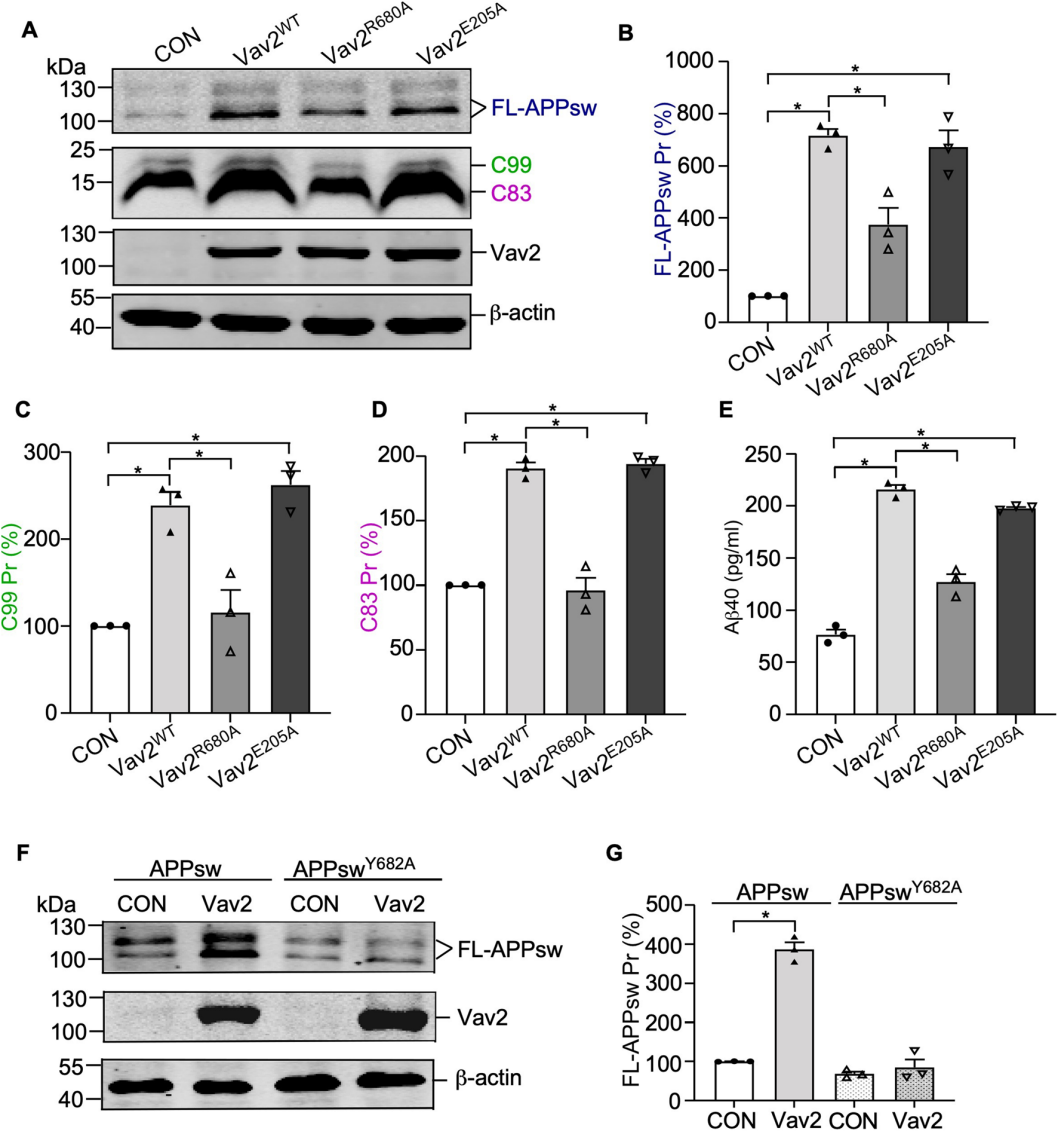
Vav2 upregulates the APP protein level and Aβ40 production. (**A**) Vav2 regulates the protein level of APP, C99 and C83 in 20E2 cells. 20E2 cells were transfected with Vav2^WT^, Vav2^R680A^, Vav2^E205A^ and empty control, respectively. Vav2, FL-APPsw, C99 and C83 protein levels were examined by Western blot, β-actin was used as loading control. Original blots are presented in Supplementary Figures. (**B**–**D**) Quantification of (**A**) using ImageJ software. All quantified results were obtained from three independent experiments. Data were presented as means ± SEM (n = 3); *p < 0.05, p values were calculated by Student’s *t* test. (**E**) Elisa was performed to measure Aβ40 in culture media from 20E2 cells transfected with Vav2^WT^ and its mutations, respectively. All quantified results were obtained from three independent experiments. Data were presented as means ± SEM.(n = 3); *p < 0.05, p values were calculated by Student’s *t* test. (**F**) HEK293 cells were co-transfected with APPsw or APPsw^Y682A^ in the presence or absence of Vav2. Western blot was performed to detect the level of FL-APPsw protein level. β-Actin was used as loading control. ImageJ software was used for quantification. Original blots are presented in Supplementary Figures. All quantified results were obtained from three independent experiments. Data were presented as means ± SEM (n = 3); *p < 0.05, p values were calculated by one-way ANOVA with Bonferroni’s multiple comparisons post hoc test.

To investigate whether Vav2 affects APP metabolism, 20E2 cells were transfected with Vav2 plasmid or empty vector (as control). 48 h after transfection, we measured the levels of FL-APPsw as well as its cleaved products C99 and C83 by western blot. As shown in Fig. 5, overexpression of Vav2 significantly increased the levels of FL-APPsw, C99 and C83 compared with control. In addition, we measured the levels of Aβ40 in the conditioned media and also observed a significant increase in the level of Aβ40 in Vav2 overexpressing cells (Fig. 5E).

Given that Vav2 binds to Y682-phosphorylated APP via its SH2 domain, we then analyzed whether a functional SH2 domain is required for Vav2 to upregulate the levels of APP and its productions. We designed a R680A mutant of Vav2 (Vav2^R680A^). As shown in Fig. 5, when cells were transfected with the mutant Vav2^R680A^, the increase of FL-APPsw and its productions was significantly diminished, compared with Vav2^WT^. This observation suggests that the SH2 domain is important for Vav2 to increase the levels of APPsw and its productions. In addition, to test whether the GEF activity of Vav2 is involved in the APPsw levels increased by Vav2 overexpression, we constructed a GEF activity-dead mutant of Vav2 (Vav2^E205A^)^57^. Similar to the overexpression of wild type Vav2, overexpression of Vav2^E205A^ mutant in 20E2 cells also led to a significant increase in the levels of APPsw and its productions, suggesting that the GEF activity is not required in this process.

To further explore whether tyrosine phosphorylation site Y682 in APP is required for its protein level elevation caused by Vav2 overexpression, we co-transfected wild type APPsw or APPsw^Y682A^ mutant with Vav2 into HEK293 cells and the levels of APPsw and APPsw^Y682A^ were measured, respectively. As shown in Fig. 5F,G, Vav2 sharply increased the protein level of APPsw, but not APPsw^Y682A^.

### Vav2 overexpression inhibits APP degradation

**Figure 6:**
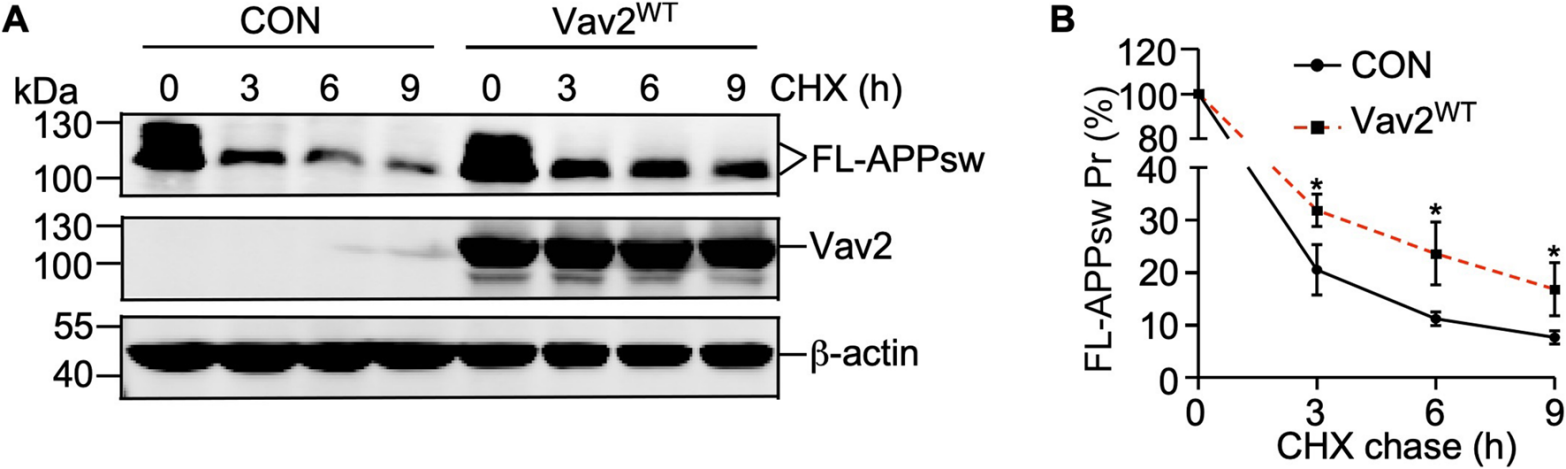
Vav2 stabilizes APP protein turnover. (**A**) Vav2 influences the degradation rate of APP protein. 20E2 cells were transfected with Vav2^WT^ or empty control. Forty-eight hours after transfection, cells were treated with 150 μg/mL of CHX for indicated times. Cell lysates were detected for FL-APPsw and Vav2 by Western blot, β-actin was used as loading control. Original blots are presented in Supplementary Figures. (**B**) Quantification of (**A**). All quantified results were obtained from three independent experiments. Data were presented as means ± SEM (n = 3); *p < 0.05, p values were calculated by Student’s *t* test at the same time point.

Next, we investigated the effects of Vav2 on APP protein degradation by cycloheximide (CHX) chase assay. 20E2 cells were transfected with Vav2 and empty control respectively and then chased with cycloheximide. In CHX chase assay, APPsw degradation rate in Vav2 transfected 20E2 cells was markedly disrupted comparing to control cells (Fig. 6A,B). These results suggest that Vav2 overexpression inhibited APP degradation.

## Discussion

In the present work, we have identified that Vav2 is a novel APP-interacting protein. It is a ubiquitous guanine nucleotide exchange factor (GEF) for Rho family GTPases. Vav2 is reported to interact with several tyrosine-phosphorylated cell surface receptors through its SH2 domain and is involved in regulating a wide range of biological processes^58,59^. The interaction between Vav2 and APP is mediated by the SH2 domain of Vav2 and the Y682 phosphorylation site in the intracellular domain of APP. Y682 has been shown to play a crucial role in modulating the binding and unbinding of APP to specific cytosolic proteins through its phosphorylation state.

Our ITC and NMR data showed that Vav2-SH2 domain can interact directly with the Y682-phosphorylated APP peptide APP-pY682. The complex structure revealed that this phosphopeptide bound to the Vav2-SH2 domain adopted a typical extended conformation. Previous study has shown that Grb2 can also bind to Y682-phosphoryled APP through its SH2 domain and the crystal structure of Grb2-SH2 in complex with Y682-phosphorylted peptide is available^29^. The reported binding Kd of Grb2-SH2 to Y682-phosphorylated peptide is 0.29 μM which is comparable to the Kd of Vav2-SH2 binding to APP-pY682, suggesting that Vav2-SH2 and Grb2-SH2 bind to Y682-phosphorylated APP with similar affinities. However, there are obvious differences in the recognition mechanism of the phosphopeptide by Vav2-SH2 and Grb2-SH2. In Grb2-SH2 complex, the phosphopeptide does not adopt an extended conformation as that seen in the Vav2-SH2 complex, but presents a folded “U” shaped structure. Both the residues Y + 2 and Y + 4 are found to contact directly with Grb-SH2. However, in the Vav2-SH2 complex, no interaction of these two residues to Vav2-SH2 domain was observed. It should be noted that although our biochemical and structural data using APP phosphopeptide has established that the Y682 phosphorylation site of APP can act as a docking site for Vav2-SH2 domain, it is unclear whether other regions of APP as well as the membrane environment would affect this interaction. Further study should be carried out using full-length APP protein under a proper model membrane system.

In this study, we have also showed that Vav2 overexpression can inhibit APP degradation and thus lead to a significantly enhancement of the levels of APP and its productions in both 20E2 cells and HEK293 cells. Using the SH2 domain mutant (R680A) and GEF-dead mutant (E205A), we found that the SH2 domain but not the GEF activity is required for Vav2 to elevate APP level. Moreover, Vav2 overexpression has no effect on the level of APPsw^Y682A^ mutant. These results may suggest a potential role of Vav2-APP interaction in the regulation of APP level.

Notably, the phosphorylation level of Y682 is significantly elevated in AD patient^18,19^. The high levels of APP Y682 phosphorylation may enhance the interaction between Vav2 and APP in AD patient. The potential role of APP-Vav2 interaction in AD need to be further studied. In addition, APP is found to be overexpressed in multiple cancers, such as breast cancer^60–62^. It has been shown recently to promote cancer cell migration and invasion^11^. However, the underline mechanism is not clear. It is well known that Vav2 is also overexpressed in most human cancers and promotes cancer cell migration and invasion in several types of human cancer^43,63–65^. Therefore, the identification of the interaction between Vav2 and APP may open up a novel avenue for further research on the role of APP in cancer.

## Methods

### Plasmid construction

For NMR and ITC experiments, the DNA fragment encoding the SH2 domain of Vav2 (residues 659–771) was cloned into a pET28a (+) (Novagen) plasmid as described previously^54^, generating a fusion protein with an N-terminus 6 × His tag. For GST pull-down experiments, the Vav2-SH2 was subcloned into pGEX4T-1 vector. The APP expression plasmid pAPPsw and the Vav2 expression plasmid pCMV5-myc-Vav2 were constructed as previously described^55,66^. Mutants were generated by PCR mediated site-directed mutagenesis. All the constructs were verified by DNA sequencing.

### Recombinant protein expression, purification and peptide synthesis

The recombinant plasmids harboring His-tagged or GST-tagged Vav2-SH2 were transformed into *Escherichia coli* BL21 (DE3) (Novagen) strain. Cells were grown at 37 °C up to an A600 nm of 0.8, and then were induced with 0.5 mM IPTG at 25 °C for 8 h. For the production of uniformly ^15^N-labeled samples, cells were grown in minimal medium using ^15^NH_4_CL (0.5 g/L) as the sole nitrogen source. ^15^N-NH_4_Cl was purchased from Cambridge Isotope Laboratories, Inc. The His-tagged proteins were purified by a chelated-nickel column followed by Thrombin protease treatment to remove the tag as previous described^54^. The GST and GST fusion proteins were purified using immobilized glutathione. All proteins were further purified on a Superdex75 gel-filtration column (GE Healthcare, Piscataway, NJ, USA). The purity of proteins was confirmed by SDS–PAGE. Protein concentrations were estimated with absorbance spectroscopy using the molar absorption coefficient.

The phosphotyrosine peptide APP-pY682 (QNG-pY-ENPT) corresponding to residues 679–686 of APP695 with Y682 phosphorylated and unphosphorylated peptide APP-Y682 (QNGYENPT) were synthesized by GL Biochem Ltd. (Shanghai).

### NMR sample preparation and NMR experiments

The NMR sample of the apo Vav2-SH2 contained 0.1 mM of protein was dissolved in NMR buffer: 20 mM Tris/HCl with 100 mM NaCl, 3 mM DTT in 90% H2O, 10% D2O (pH 7.0). D2O was purchased from Cambridge Isotope Laboratories, Inc. For the SH2/peptide complex samples, the ^15^N-labeled Vav2-SH2 (0.1 mM) mixed with phosphotyrosine peptide APP-pY682 (8 mM) or APP-Y682 (8 mM) which were dissolved in the same NMR buffer and the solutions were adjusted to pH 7.0 using NaOH. A series of ^1^H–^15^N HSQC spectra were recorded as the titrant gradually titrated into the protein solutions using the Bruker hsqcfpf3gpphwg pulse program. NMR experiments were carried out at 293 K on a Bruker Avance 600 MHz NMR spectrometer equipped with cryoprobes. NMR data were processed with NMRPipe^67^ and analyzed using Sparky3 (Goddard and Kneller, University of California, San Francisco). The assignment of Vav2-SH2 was extracted from a previous study^55^.

### Isothermal titration calorimetry (ITC)

ITC experiments were performed at 298 K on a PEAQ-ITC titration calorimeter (MicroCal, Northampton, MA). Vav2-SH2 domain wild type and mutants were dialyzed extensively against the titration buffer containing 10 mM Tris/HCl, 100 mM NaCl (pH 7.4). Peptides were dissolved in the same buffer and the solutions were adjusted to pH 7.4 using NaOH. 0.75 mM peptides were titrated into Vav2-SH2 domain wild type or its mutant (30 μM). Control experiments were performed under the same condition by titrating same peptide into buffer alone. ITC data were analyzed with a single-site binding model using Microcal PEAQ-ITC software.

### Protein crystallization

For crystallization experiments, purified Vav2-SH2 protein was concentrated to 15 mg/mL in a buffer containing 10 mM Tris/HCl, pH 7.4, 100 mM NaCl and 5 mM β-mercaptoethanol. Purified protein was mixed with pY682 peptide at a 1:1.2 molar ratio and crystallized using the sitting-drop vapour-diffusion method at 16 °C after mixing 0.5 μL of the protein solution with 0.5 μL of the reservoir solution. Crystals were obtained with a reservoir solution containing 25% PEG 3350, 0.2 M MgCl_2_·6H_2_O and 0.1 M Tris/HCl, pH 8.4. Before flash-freezing crystals in liquid nitrogen, crystals were soaked in a cryoprotectant consisting of 85% reservoir solution and 15% glycerol.

### Data collection and structure determination

X-ray diffraction data were collected at the Shanghai Synchrotron Radiation Facility (SSRF) beam line BL18U. The data were processed with HKL2000^68^ and programs in the CCP4 suite^69^. The structure of the Vav2-SH2 domain complexed with pY682 was solved by molecular replacement with co-ordinates from PDB entry 3MXC^29^ and the program PHASER^70^. All the structural models were subsequently refined by programs REFMAC5^71^, PHENIX^72^, and COOT^73^. Crystallographic parameters are listed in Table 1. All structure figures were prepared with PyMOL. The transition state complex interface was calculated in PDBePISA.

### Cell cultures, transfection

HEK293 cells were maintained in Dulbecco’s modified Eagle’s medium supplemented with 10% (V/V) fetal calf serum and penicillin/streptomycin. The 20E2 cell line, a Swedish mutant APP stable HEK293 cell line was maintained in complete DMEM supplemented with 100 μg/mL Zeocin. Cells were transfected using Lipofectamine™ 3000 (Invitrogen) reagent according to the manufacturers’ protocols.

### Western blotting and antibodies

For immunoblotting analyses, 20E2 cells were lysed in RIPA lysis buffer supplemented with protease and phosphatase inhibitors (Roche Applied Science). Immunoblotting was performed as described previously^74^. Primary antibodies used are: anti-myc tag mAb (Cell Signaling, Danvers, MA), C20 antibody, anti-β actin mAb (Sigma-Aldrich), anti-Phospho-Tyrosine mAb (Cell Signaling, Danvers, MA). Detection was performed with the Li-Cor Odyssey imaging system and quantitated with ImageJ software.

### GST pull-down assays

One hundred microliter glutathione-agarose beads were incubated with 1 mg purified GST or indicated GST-SH2 fusions and the mutant GST-SH2^R680A^ for 2 h at 4 °C in GST binding buffer (20 mM Tris/HCl, pH 7.4, 150 mM NaCl and 1 mM EDTA, 0.5% TritonX-100). The beads were then washed 4 times with GST binding buffer and incubated for another 2 h with 0.2 mg indicated lysates of 20E2 cells stable expressing the indicated APPsw constructs. After washing 4 times with GST binding buffer, beads were boiled in SDS sample buffer, run on 12% SDS–PAGE gel and analyzed by Coomassie or immunoblotting.

### Co-immunoprecipitation (co-IP)

HEK293 cells were co-transfected with pAPPsw and pCMV5-myc-Vav2. For co-IP, cells were harvested after 48 h and lysed in 1 mL of 1% NP-40 lysis buffer supplemented with protease and phosphatase inhibitors (Roche Applied Science). Cell lysates were then incubated with primary antibody and protein A/G-agarose beads (Santa Cruz Biotechnology, Santa Cruz, CA) at 4 °C overnight. Mouse or rabbit IgG (Beyotime Institute of Biotechnology, Haimen, China) was performed with protein A/G-agarose beads as negative controls. After washing 3 times with PBS, beads were boiled in SDS sample buffer, run on 8% SDS–PAGE gel and analyzed by immunoblotting with indicated antibodies.

### Immunofluorescence

Immunofluorescence was performed as previously described^75^. Primary antibodies used were C20 antibody and anti-myc tag mAb (Cell Signaling, Danvers, MA). Secondary antibodies were CoraLite488-conjugated Affinipure Goat Anti-Rabbit IgG and CoraLite594-conjugated Affinipure Goat Anti-Mouse IgG (proteintech, Wuhan, China). Images were captured by LSM 880 fluorescent microscope (Carl Zeiss, Jena, Germany) and analyzed with ZEN software.

### Elisa

20E2 cells were transfected with Vav2^WT^, Vav2^R680A^, Vav2^E205A^ and empty control, respectively. Forty-eight hours after transfection, the level of Aβ40 in the supernatant was measured using the Aβ1–40 Elisa kit (Cloud-Clone Corp, Wuhan, China) according to the manufacturer’s protocol.

### Cycloheximide (CHX) pulse-chase assay

The 20E2 cells were transfected with Vav2^WT^ and empty control, respectively. Forty-eight hours after transfection, cells were treated with 150 μg/mL of CHX (MCE, Shanghai, China) and harvested after 0, 3, 6 and 9 h, respectively. The protein level of FL-APPsw was detected by western blotting and quantitated with ImageJ software.

### Data analysis

Data are presented as means ± SEM from three to five independent experiments. Student’s *t* test was performed for differences between two groups. One-way or two-way ANOVA with Bonferroni’s multiple comparisons post hoc test was applied for multigroup comparisons. All analyses were performed with GraphPad Prism 9 software (GraphPad). Differences were defined to be statistically significant at p < 0.05.

## Supplementary Information

Supplementary Information.

## Data Availability

Raw data is available from the corresponding authors upon reasonable request. Structure data are deposited in the Protein Data Bank with the accession code 7WFY.
