# Supplementary material for: Vav2 is a novel APP-interacting protein that regulates APP protein level
Source: Sci Rep. 2022 Jul 26;12:12752. doi: 10.1038/s41598-022-16883-z (PMC9325707; doi:10.1038/s41598-022-16883-z)

Vav2 is a novel APP-interacting protein that regulates APP protein level

Youjia Zhang^1,2,#^, Xiaxin Yang^3,4,#^, Yongrui Liu^2^, Liang Ge^1^, Jiarong Wang^1^, Xiulian Sun^4,^^5^^,6,*^, Bo Wu^1,*^ and Junfeng Wang^1,2,7,*^

**
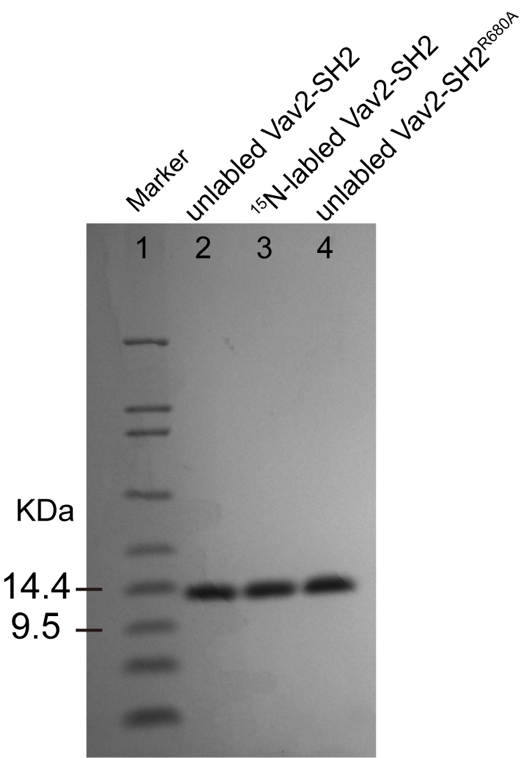
Figure S1. SDS-PAGE analysis of the purified Vav2-SH2 proteins.**


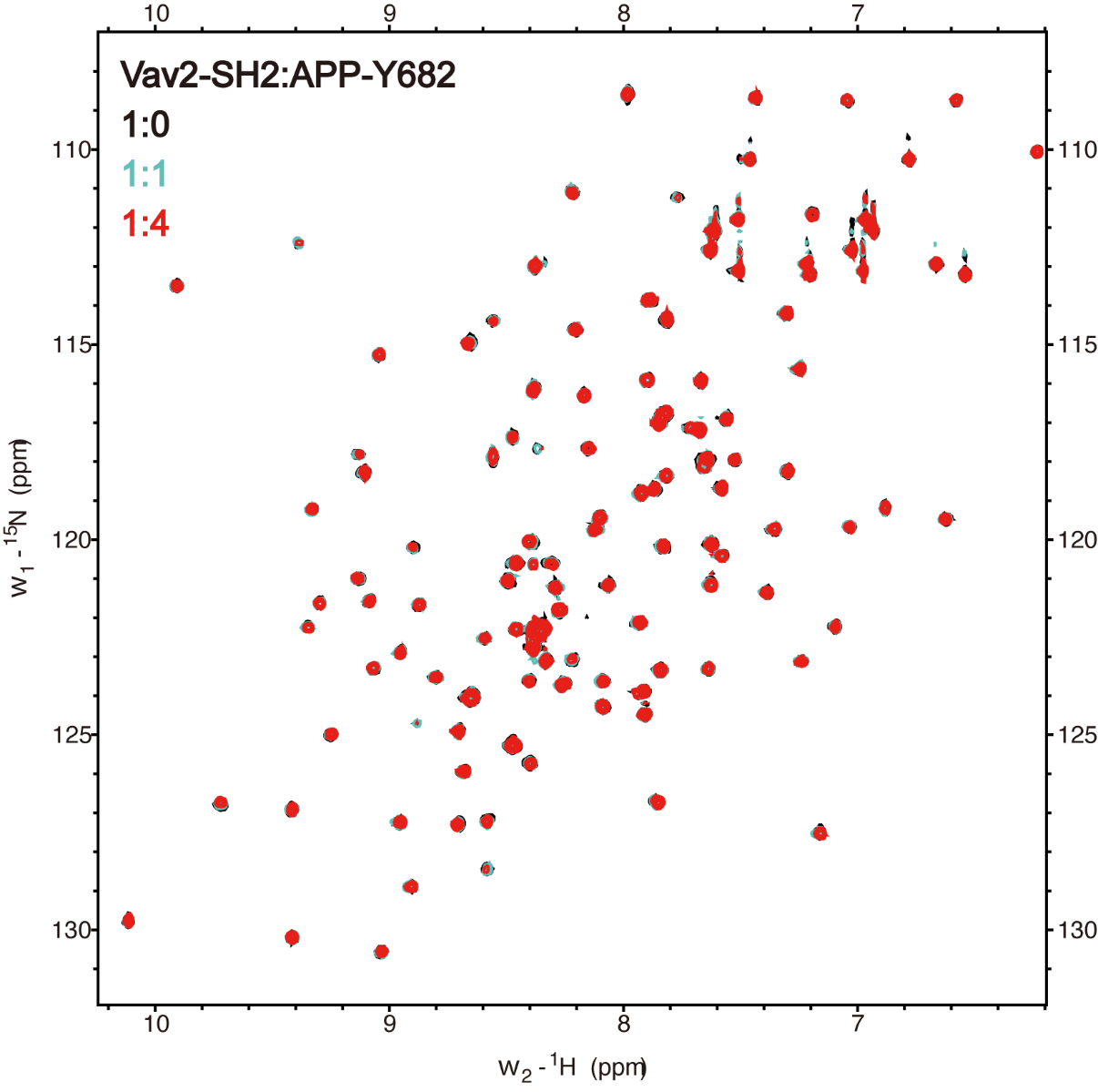


**Figure S2. NMR titration of Vav2-SH2 with unphosphorylated** **peptide** **APP-Y682.** Superimposition of the ^1^H–^15^N HSQC spectra of the Vav2-SH2 titrated with APP-Y682 at a molar ratio of 1:0 (black), 1:1 (cyan) and 1:4 (red).


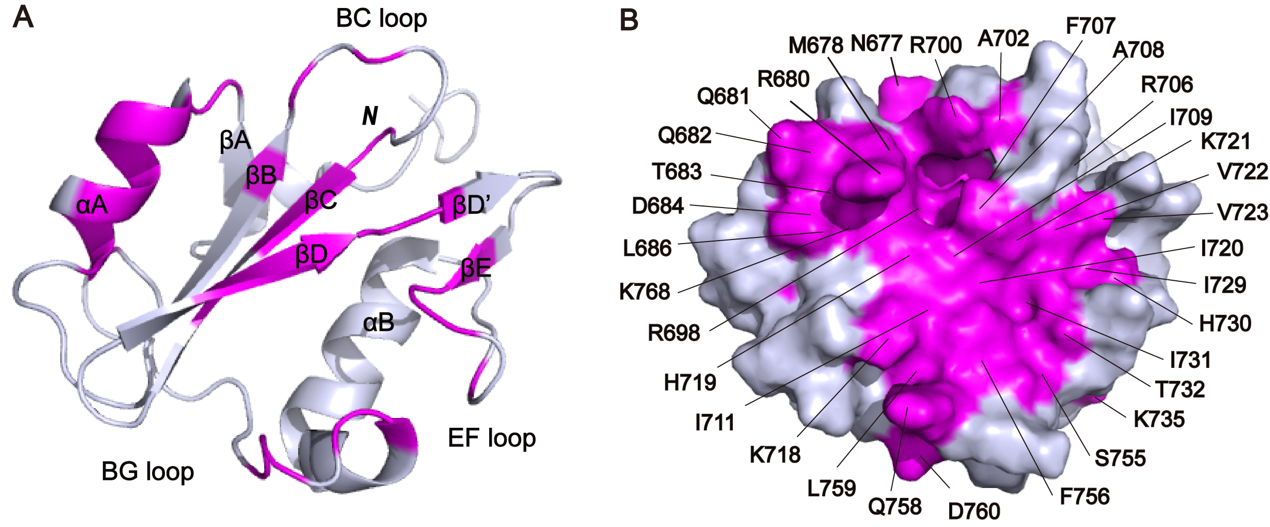


**Figure S3. Binding of Vav2–SH2 to Y682-phosphorylated APP peptide.**

1. and (B) The cartoon (A) and surface (B) representations of Vav2–SH2 structure with the significantly perturbed residues upon binding to the peptide APP-pY682 are colored and labeled.


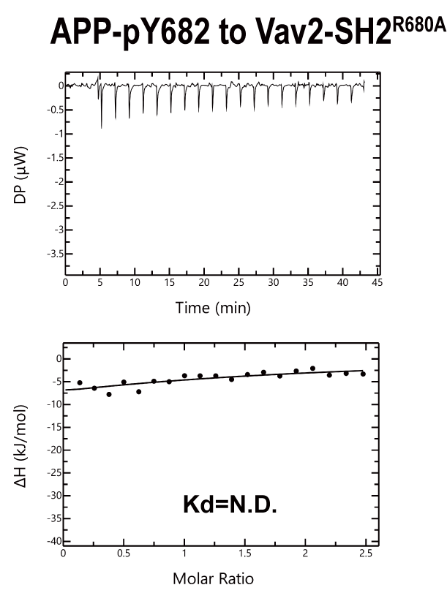


Figure S4. **ITC measurements of the binding affinity of APP-pY682 peptide to the Vav2-SH2^R680A^ mutant.**


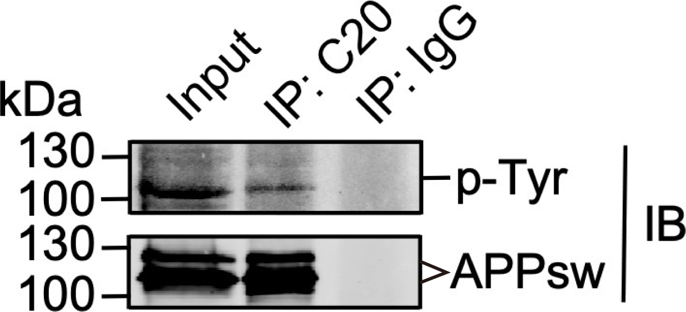


**Figure S5.** **IP was performed using 20E2 cells.** C20 was used as the pull-down antibody and anti-Phospho-Tyrosine antibody was used as detection antibody. Original blots are presented in Supplementary Figures.

**Table S1**

**Full amino acid sequences for the both peptide and protein**

| **protein /peptide** | **Full amino acid sequence** |
| --- | --- |
| Vav2-SH2 | GSHM^659^SRPPSREIDYTAYPWFAGNMERQQTDNLLKSHASGTYLIRERPAEAERFAISIKFNDEVKHIKVVEKDNWIHITEAKKFDSLLELVEYYQCHSLKESFKQLDTTLKYPYKSRE^771^ (The N-terminal GSHM are residues left after thrombin protease cleavage) |
| APP-Y682A | QNGYENPT |
| APP-pY682 | QNG-pY-ENPT |

**Raw Data Images**


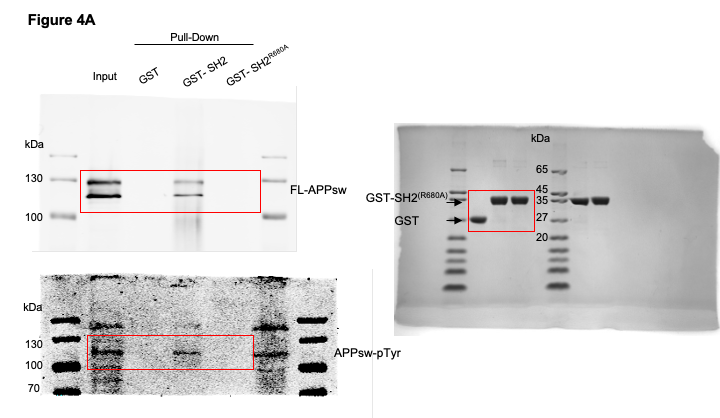
**Of Figure 4A in the main text:** Western blots were cut prior to antibody hybridization. SDS-PAGE from Figure 4A was imaged as full-length Coomassie blue staining.


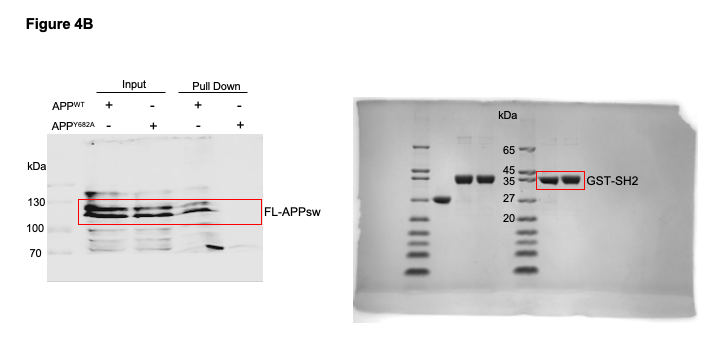
**Of Figure 4B in the main text:** Western blots were cut prior to antibody hybridization. SDS-PAGE from Figure 4A was imaged as full-length Coomassie blue staining.

**
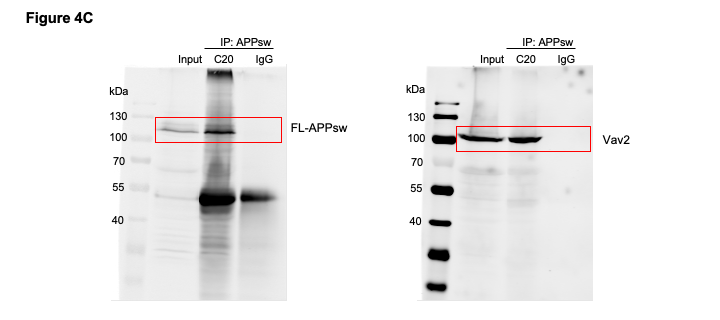
Of Figure 4C in the main text:** Western blots were cut prior to antibody hybridization.


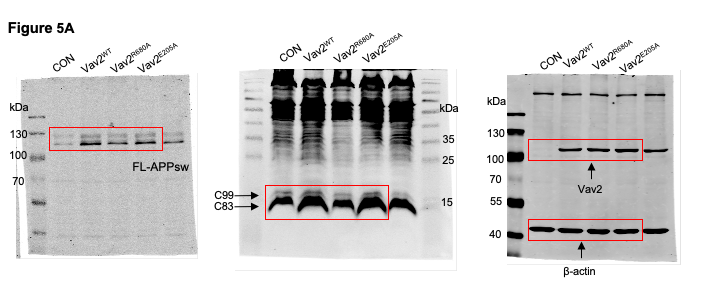
**Of Figure 5A in the main text:** Western blots were cut prior to antibody hybridization.


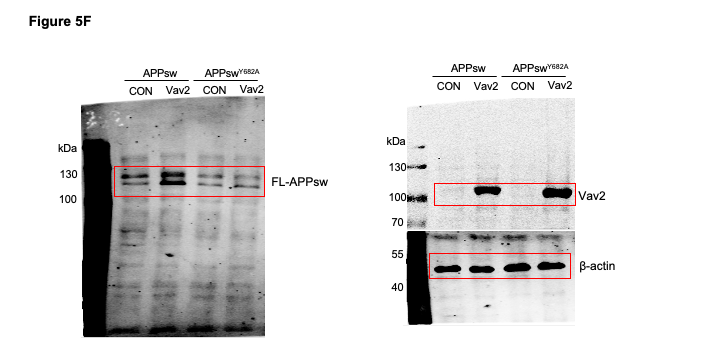
**Of Figure 5F in the main text:** Western blots were cut prior to antibody hybridization.


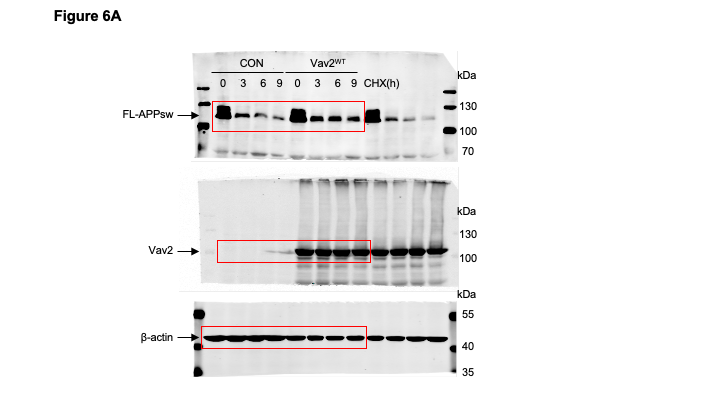
**Of Figure 6A in the main text:** Western blots were cut prior to antibody hybridization.

**Of Figure S5 in the supplementary data:** Western blots were cut prior to antibody hybridization.


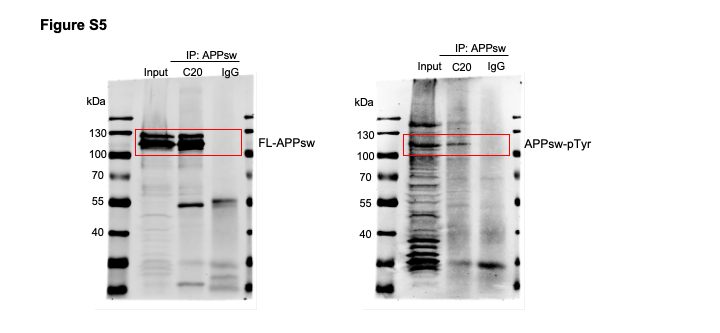

Supplement: Supplementary file 1 — Supplementary Information. [file 41598_2022_16883_MOESM1_ESM.docx]
